# Polyethylene Glycol Ointment Alleviates Psoriasis-Like Inflammation Through Down-Regulating the Function of Th17 Cells and MDSCs

**DOI:** 10.3389/fmed.2020.560579

**Published:** 2021-03-22

**Authors:** Yan Lu, Yi Xiao, Ming-Zhu Yin, Xing-Chen Zhou, Li-Sha Wu, Wang-Qing Chen, Yan Luo, Ye-Hong Kuang, Wu Zhu

**Affiliations:** ^1^Department of Dermatology, Xiangya Hospital, Central South University, Changsha, China; ^2^Hunan Key Laboratory of Skin Cancer and Psoriasis, Xiangya Hospital, Central South University, Changsha, China; ^3^Pharmacy, Xiangya Hospital, Central South University, Changsha, China; ^4^Institute of Medical Science, Xiangya Hospital, Central South University, Changsha, China

**Keywords:** psoriasis, IMQ, PEG, MDSCs, Th17

## Abstract

**Objective:** To explore the possible mechanism of improving the imiquimod (IMQ)-induced psoriasis-like inflammation by using polyethylene glycol (PEG) ointment.

**Methods:** We evaluated the appearance of psoriasis lesions by Psoriasis Area and Severity Index (PASI), observed the epidermal proliferation by histopathological staining and immunohistochemical staining, and explored the key molecules and signaling pathways of improving psoriasis-like inflammation treated with PEG ointment by RNA sequencing. Finally, we verified the expression of inflammatory cells and inflammatory factors by flow cytometry, immunohistochemical staining, and Q-PCR.

**Results:** PEG ointment could improve the appearance of psoriasis lesions and the epidermis thickness of psoriasis mouse, inhibit the proliferation of keratinocytes, and down-regulate the relative mRNA levels of IL-23, IL-22, IL-6, IL-17C, IL-17F, S100A7, S100A8, S100A9, CXCL1, CXCL2, and IL-1β in the skin lesions of psoriasis mouse by down-regulating the numbers of myeloid-derived suppressor cells (MDSCs) and T helper 17 (Th17) cells.

**Conclusion:** PEG ointment could improve the IMQ-induced psoriasis-like inflammation by down-regulating the functions of Th17 cells and MDSCs.

## Introduction

Psoriasis is a chronic autoimmune disease affecting 0.09 to 5.1% of the general population worldwide (1–4). The mechanism of psoriasis is very complicated and has not been fully elucidated. Given that ~80% of the patients with psoriasis are in mild-to-moderate states, topical physiotherapy can be used to control the condition, and therefore, the development of external medicine is the preferred option. Imiquimod (IMQ) is a ligand for Toll-like receptors (TLR7 and TLR8). IMQ has been used for the topical treatment of genital and perianal warts caused by human papilloma virus (5); unexpectedly, the patients who suffered from genital and perianal warts developed psoriasis-like lesions at the affected areas after applying IMQ cream (6, 7). It has been proven that the IMQ-induced psoriasis-like skin lesions in mice, such as erythema, skin thickening, scaling, epidermal alteration (acanthosis, parakeratosis), inflammatory cell infiltration, and vascular proliferation, are very similar to psoriasis (8–10). The psoriasis mouse model is widely used in the screening of topical drugs for psoriasis. There have been an increasing number of studies in recent years focusing on the treatment of psoriasis using topical medicine, and the drugs that are currently being studied mainly concentrate on non-toxic and harmless natural Chinese herbal medicine extracts with anti-inflammatory and anti-proliferative effects (11). In our study, it was found that polyethylene glycol (PEG) (referring to a polymer or oligomer of ethylene oxide) as an ointment base could significantly improve the IMQ-induced psoriasis-like inflammation. PEGs with a molecular weight of 200–600 are in a liquid state at room temperature, while those with a molecular weight of >600 gradually enter a semi-solid state. PEGs with different molecular weights usually have different physical properties and applications, ranging from colorless, odorless viscous liquids to waxy solids. Most of the PEG chemistries are similar. PEGs with low molecular weights can be used as suppository base, eye drops, injections, and solvents, while PEGs with high molecular weights can be used for preparing tablets and film coats. Liquid and solid PEGs can be used as suppository base and ointment base at various ratios. Therefore, PEG has been widely used in the cosmetics and pharmaceutical industries. However, there was no report yet focusing on the therapeutic mechanism of PEG. In this study, we found that PEG ointment could improve the IMQ-induced psoriatic-like inflammation and epidermal keratinocyte proliferation, and we explored the possible mechanism of PEGs in the treatment of psoriasis by applying the RNA-seq method.

## Materials and Methods

### Preparation of PEG Ointment

The PEG ointment is composed of 70% of PEG400 (SCRC, shanghai, China) and 30% of PEG4000 (SCRC, shanghai, China). Mix the PEG400 and PEG4000 ingredients and stir until well-distributed at 65°C, and then stir into a semi-solid state at room temperature.

### Animals and Ethical Statement

A total of 18 male BALB/c mice (8 weeks) were purchased from Hunan Slack King Experimental Animal Co. During the experiment, mice were fed under specific pathogen-free conditions with controlled environmental conditions (12 h light/dark cycle, at 24°C) and provided with standard water and food *ad libitum*. All animal experiments were performed according to the principles specified in the “Guide for the Care and Use of Laboratory Animals in China” with approval from the Animal Ethics Committee of Central South University.

### Animal Treatment

Mice at 8 to 11 weeks of age received a daily topical dose of 62.5 mg of commercially available IMQ cream (5%) (Aldara; 3M Pharmaceuticals) on the shaved back for 5 or 6 consecutive days to build a psoriasis mouse model. Besides, we can also extend the 6-day psoriasis model to 21 days by continuing to apply 62.5 mg of IMQ cream once every other day for 14 consecutive days (12, 13).

The 18 mice were randomly divided into three groups (*n* = 6): the Blank group, the IMQ group, and the PEG group. All mice were marked on the tail, and an area of 2 cm × 3 cm was shaved from the back of the mice. The mice in the Blank group were not given any intervention; the mice in the IMQ group were only applied with IMQ cream (62.5 mg/day); and the mice in the PEG group were applied with IMQ cream (62.5 mg/day) first, and then with PEG ointment (100 mg/day) 6 h later. The experimental period lasted for 6 days or 20 days. For the establishment of the 21-day psoriasis mouse models, 62.5 mg of IMQ cream was applied to each mouse in the IMG group once a day during the first 6 days and once every 2 days from day 7 to day 20; 100 mg of PEG ointment was applied to each mouse in the PEG group every day from day 1 to day 20 after applying IMQ cream. At the end point of the experiment, all the mice were weighed and sacrificed by breaking the neck. Skin lesions and spleens were collected for future investigation.

### Scoring Severity of Skin Inflammation

An objective scoring system [Psoriasis Area and Severity Index (PASI)] was used to evaluate the severity of skin inflammation of the mouse model. Erythema, scaling, and infiltration were scored independently from 0 to 4 as follows: 0, none; 1, slight; 2, moderate; 3, marked; 4, very marked. The cumulative score (erythema plus scaling plus thickening) was calculated to indicate the severity of inflammation (scale 0–12).

### Histology and Immunohistochemistry

The skin lesions from each mouse were fixed in 4% paraformaldehyde and then embedded in paraffin for 24–36 h. Sections (thickness 4 μm) were stained using hematoxylin and eosin (H&E). The tissue pathology was observed under a light microscope (Nikon, Beijing, China). As for immunohistochemistry, the section slides were heated at 60°C for 2 h first, and washed in xylene and hydrated with varying concentrations of alcohol. After repairing the antigen through high temperature addition, the sections were incubated in rabbit anti-Ki67 (Abcam, ab16667, USA) and rabbit anti-IL-17a (Seivicebio, GB11110, China) overnight at 4°C. Then, the secondary antibody was applied to detect the primary antibody following the manufacturer's instructions of the immunohistochemical general two-step test kit. Subsequently, the section slides were stained with DAB chromogen and counterstained with hematoxylin. The tissue pathology was observed under a light microscope (Nikon, Beijing, China).

### Changes of Spleen in Mice

The mice were weighed before being sacrificed. Then, each spleen was weighed, and the spleen index was calculated accordingly [spleen index = 100 ^*^ spleen weight (mg)/body weight (g)].

### Flow Cytometric Cell Staining

The mouse spleen was isolated and ground, and the red blood cells were lysed with 1 × lysing buffer (BD, USA). Then, the spleen cells were resuspended with 1× PBS buffer for staining. The following Abs were used for surface staining: anti-mouse CD45-FITC (Biolegend, USA), anti-mouse CD4-PerCP/Cy5.5 (Biolegend, USA), anti-mouse CD11b-PE (Biolegend, USA), and anti-mouse Gr-1-FITC (Biolegend, USA). The anti-mouse IL-17-PE was used for intracellular staining. For reconstitution, pre-warm the kit to room temperature; add 100 μl of DMSO to one vial of Zombie Aqua™ dye (Biolegend, USA) and mix until fully dissolved. Dilute Zombie Aqua™ dye at 1:500 in PBS. Resuspend 10 × 10^6^ cells in 100 μl of diluted Zombie Aqua™ solution. Incubate for 10–15 min at RT away from light. Add the cell surface antibody cocktail without washing the cells and incubate for another 30 min at 4°C away from light. Next, wash the cells with 1× PBS buffer. Last, stain the cells simultaneously with 4% paraformaldehyde for detection by flow cytometry. As for the intracellular staining of T helper 17 (Th17) cells, first cells were cultivated with 100 μl of PBS including 25 ng/ml of PMA, 1 μg/ml of ionomycin, and 10 μg/ml of brefeldin A for 6 h at 37°C away from light. Then, the cells were stained with the surface antibody mix (CD45/CD4) for 30 min at 4°C away from light and suspended with Fixation/Permeabilization solution for 30 min at 4°C away from light. Last, the cells were stained with the intracellular antibody for 30 min at 4°C away from light and detected by flow cytometry (FACSCalibur, BD, San Jose, CA).

### Quantitative PCR

PCR was performed after reverse-transcribing the RNA with HiScript Q RT SuperMix for qPCR (Vazyme, R123-01, NanJing) by a Veriti 96-well Thermal cycler (Applied Biosystems) using the TaqMan primer sets purchased from Sangon Biotech. The sequences of these primers are shown in Supplementary Table 1. All values were normalized to the expression of the housekeeping gene GAPDH. Statistical significances were observed between Blank, IMQ, and PEG groups.

### Transcriptomic Analysis

For microarray analysis, the gene expression profiles were generated using customized 4 x 44 k oligonucleotide microarrays (Agilent Technologies). Sample preparation, labeling, and hybridization were performed according to the manufacturer's protocol. The microarray expression profiles were generated using Agilent's Feature Extraction software (Version 9.5.1). For RNA sequencing, the Dynabeads® mRNA Purification Kit (Invitrogen) was used to purify mRNA from total RNA, and ERCC RNA spike-in was added according to the user guide. Then, library construction was performed according to the non-stranded TruSeqs™ protocol, and clusters were generated according to the TruSeq PE cluster Kit v3 reagent preparation guide (for cBot-HiSeq/HiScanSQ). The high-throughput shotgun sequencing was performed on the Illumina HiSeq 2000 platform. Paired-end reads with lengths of 90 and 100 nucleotides were generated for 12 samples and 486 samples, respectively.

### Statistical Analyses

All experiments were repeated three times at least. Statistical analyses were carried out using the unpaired two-tailed Student's *t* test and one-way analysis of variance (ANOVA). Statistical significance was accepted at the level of *P* < 0.05. Photographs were captured and processed using GraphPad Prism 5 software and Adobe Photoshop CS5 software. All the statistical analyses were performed using GraphPad Prism 5 software and IBM SPSS Statistics 23 software.

## Results

### PEG Ointment Improves the Appearance of IMQ-Induced Psoriasis-Like Lesions

**Figure 1:**
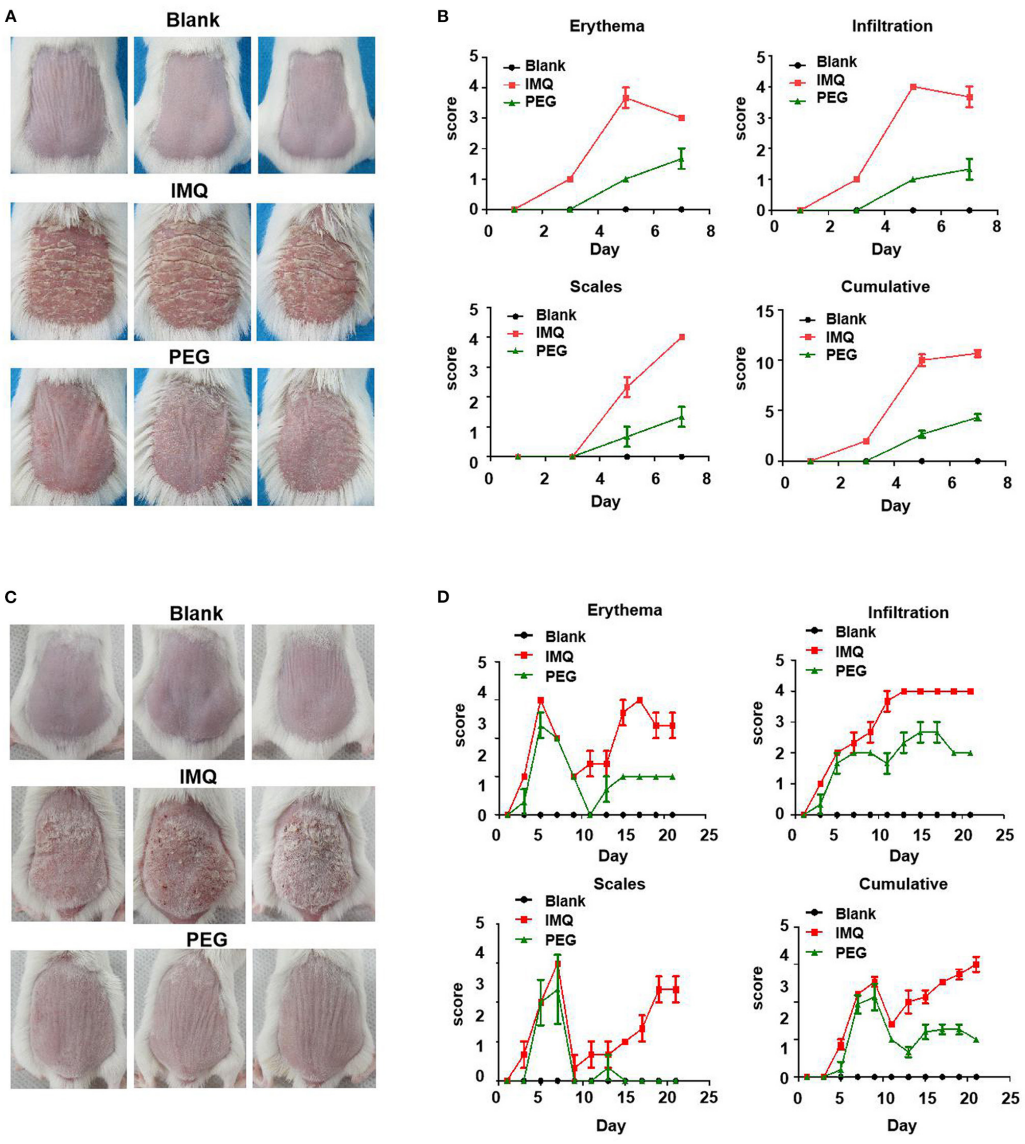
Skin lesion appearance of the IMQ-induced psoriasis mouse model, PASI score graph. **(A)** At the end point of the psoriasis mouse model for 7 days, before all mice were killed, photos had been taken, with six mice in each group, and three images were selected for each group. **(B)** Scores of erythema, scales, and infiltration of skin lesions in each group at different time points. **(C)** At the end point of the psoriasis mouse model for 21 days, before all mice were killed, photos had been taken, with six mice in each group. **(D)** Scores of erythema, scales, and inltration of skin lesions in each group at different time pointsand three images were selected for each group. We have repeated the experiment more than three times.

The skin lesions of the IMQ-induced psoriasis mouse model exhibited the following symptoms: erythema, scales, and infiltration. The effect of PEG ointment on psoriasis mice was observed. In the two different duration models, the back skin of IMQ mice showed obvious erythema, scales, and infiltration, indicating that we successfully induced a psoriasis mouse model. The PASI scores of the lesions were scored during the experiment. At the end of the experiment, the mice were photographed using a camera to record the appearance of the lesion (Figures 1A,C). Figure 1A shows the 7-day model, and Figure 1C shows the 21-day model. We found that IMQ mice showed obvious erythema, scales, and infiltration, However, the PEG group showed slight erythema, scales, and infiltration, indicating that the psoriasis-like inflammation was significantly alleviated after treatment with PEG ointment. The analysis of PASI score was shown in Figures 1B,D. During the psoriasis mouse model for 6 days (Figures 1A,B), we observed that the erythema and infiltration scores of the psoriasis mice increased rapidly from day 3, reached the peak on the 5th day, and then slightly decreased on the 7th day. The scales turned up from day 3 and reached the peak on the 7th day. Meanwhile, the 21-day psoriasis mouse model also demonstrated that PEG ointment could improve the appearance of the IMQ-induced psoriasis-like lesions (Figures 1C,D), and we found that the PASI score experienced two cycles of exacerbation-reduction-heaviness of skin lesions.

### PEG Ointment Down-Regulates the High Expression of Ki67 in Epidermis

**Figure 2:**
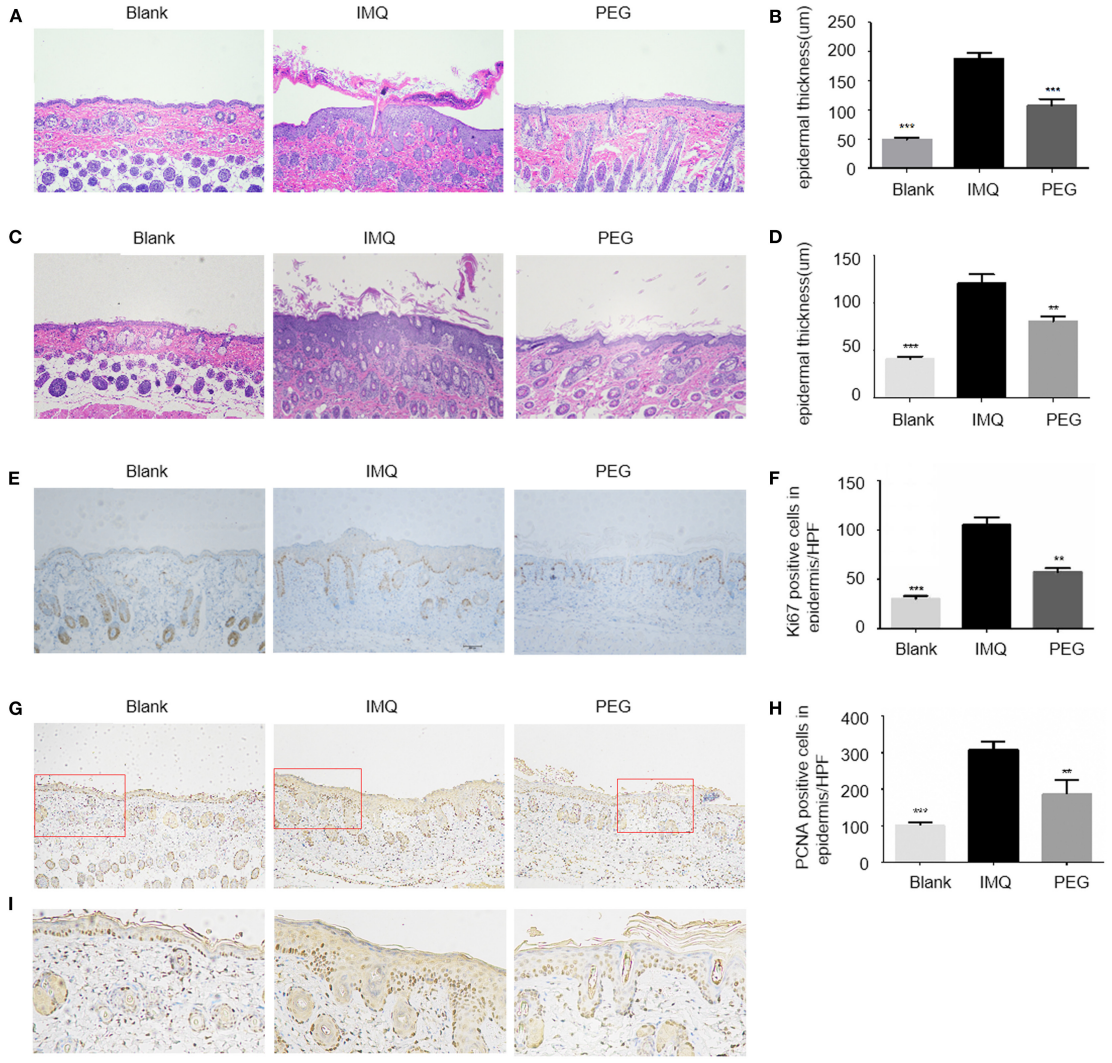
**(A,C)** HE staining of lesion, with one representative for each group. **(A)** HE staining result of the 7-day model (100 ×). **(B)** The columnar statistical graph of the epidermal thickness of each group of the 7-day model (*compared with the IMQ group, ***P < 0.001). **(C)** HE staining result of the 21-day model (100 ×). **(D)** The columnar statistical graph of the epidermal thickness of each group of the 21-day model (*compared with the IMQ group, **P < 0.01, ***P < 0.001). **(E)** The immunohistochemistry staining of Ki67. It can be seen that the brown particles in the basal part of the epidermis are Ki67-positive expression (100 ×). **(F)** A bar graph of the number of Ki67-positive expressions/HPF in the basal part of the epidermis (*compared to the IMQ group, **P < 0.05, ***P < 0.001). **(G)** The immunohistochemistry staining of PCNA. It can be seen that the brown particles in epidermis are PCNA-positive expression (100 ×). **(H)** A bar graph of the number of PCNA-positive expressions/HPF in epidermis (*compared to the IMQ group, **P < 0.05, ***P < 0.001). **(I)** A partial enlarged view of PCNA staining from the red box in **(G)**. All statistical graph and data analysis were finished by GraphPad Prism 5 software. We have repeated the experiment more than three times.

The main pathological manifestations of lesions in psoriasis patients are hyperkeratosis with parakeratosis, and the pathological tissue of the psoriasis mouse model has similar manifestations. At the end of the experiment, the lesions were collected, fixed, and stained using the H&E method (Figures 2A–D). The results showed that the epidermal thicknesses of the mice in the IMQ group were significantly increased compared with that in the Blank group, while the epidermal thicknesses of the mice in the PEG group were significantly reduced compared with that in the IMQ group. The immunohistochemical staining of the epidermal basal nucleus Anti-Ki67 antibody and Anti-PCNA was used to compare the proliferation of keratinocytes among three groups (Figures 2E,F). There were a larger number of brown Ki67-positive cells in the basal part and PCNA-positive cells of the epidermis of the mice in the IMQ group; however, the number of brown Ki67-positive cells and PCNA-positive cells was less in the PEG group than in the IMQ group. It can be seen from Figures 2G,H that PEG ointment significantly down-regulated the high expression of Ki67 and PCNA in the psoriasis mice induced by IMQ cream. In order to facilitate observation, we zoomed in on the partial staining of PCNA in the red box (Figure 2I).

### Differentially Expressed Genes

**Figure 3:**
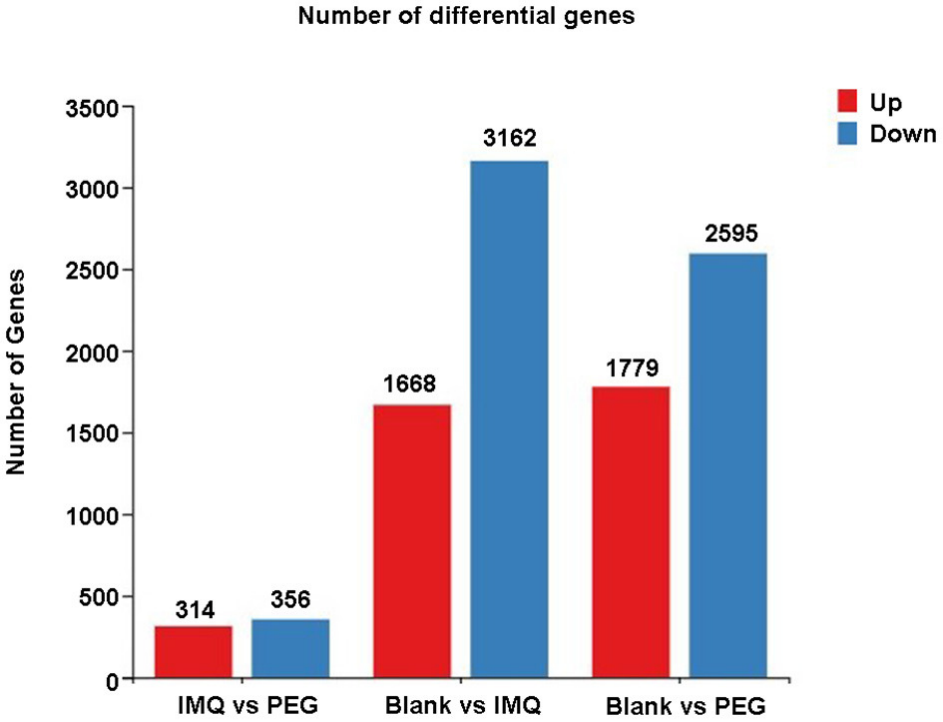
A statistical graph of differential gene expression among the three groups. Red represents up-regulated genes and blue represents down-regulated genes.

**Figure 4:**
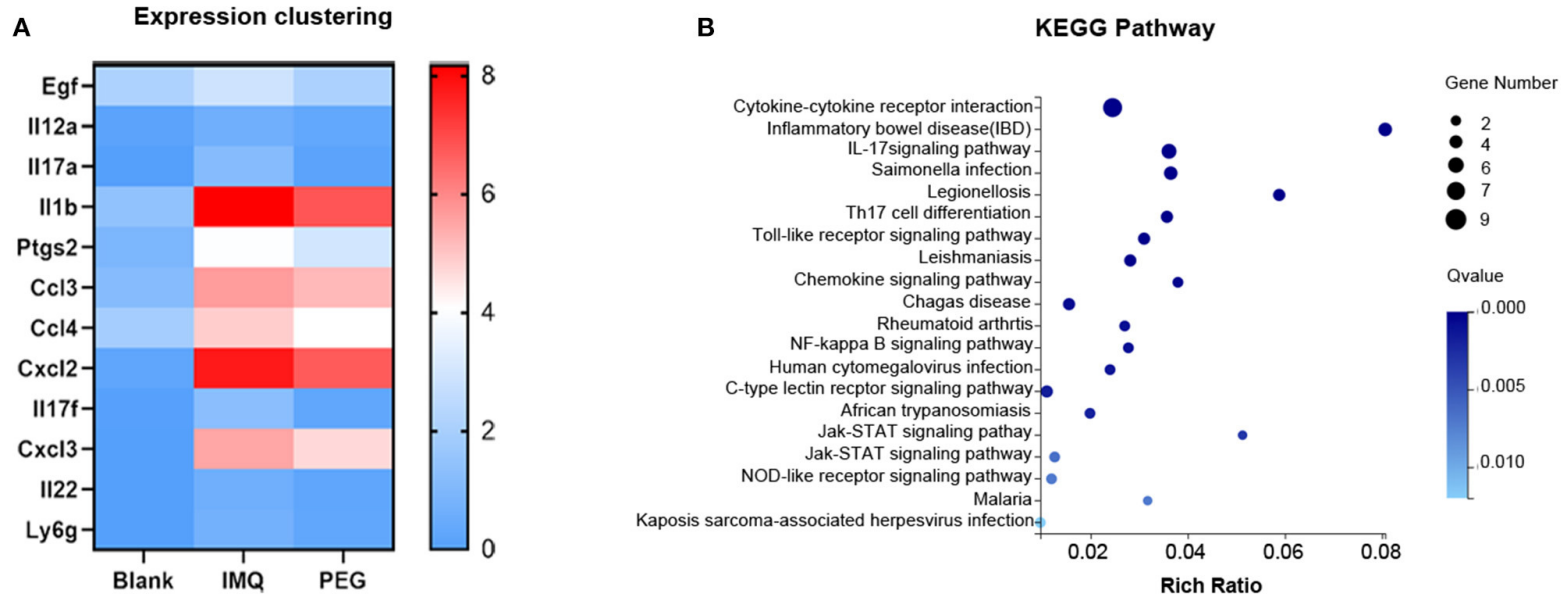
**(A)** Expression clustering heat map; the horizontal axis represents the log2 (FPKM+1) of the sample, and the vertical axis represents the gene. Under the default color scheme, the redder the color, the higher the expression level, and the bluer the color, the lower the expression level. **(B)** KEGG Pathway enriches the bubble chart; the *X*-axis is the enrichment ratio, and the *Y*-axis is the KEGG Pathway. The size of the bubble indicates the number of genes annotated to a KEGG Pathway. The color represents the enriched *Q* value. The darker the color, the smaller the *Q* value.

From the results above, it can be seen that PEG ointment could significantly improve the IMQ-induced psoriasis-like inflammation. However, the underlying mechanism has not been fully elucidated, so we performed RNA sequencing to discover the differential genes in skin lesions. It was found that 314 genes were up-regulated and 356 genes were down-regulated in the PEG group compared with the IMQ group (Figure 3). Then, we selected 12 inflammation-related genes (Table 1) for cluster analysis and KEGG Pathway enrichment analysis from the 356 down-regulated genes (Figure 4) and found that these differential genes mainly concentrated on the “Cytokine–Cytokine receptor interaction,” “IL-17 signaling pathway,” “Th17 cell differentiation,” and “Chemokine signaling pathway.” Given that the pathogenesis of psoriasis is closely related to the IL23/Th17 axis, we focused on the IL-17 signaling pathway. Besides, the gene Ly6g is the marker of myeloid-derived suppressor cells (MDSCs), and MDSCs can regulate the Th17 cell differentiation by IL-1β, so we also followed MDSCs.

**Table 1:** 12 candidate genes and information.

**Gene**	**IMQFPKM**	**PEGFPKM**	**log2 (PEG/IMQ)**	***P*-value (IMQ vs. PEG)**
Cc13	145.436	66.603	−1.110483116	1.17E-257
Cc14	76.57	27.51	−1.444101609	3.47E-172
Cxcl2	491.093	212.14	−1.19719882	0
Cxcl3	126	59.626	−1.064298513	1.72E-266
Egf	6.98	3.173	−1.157795634	1.25E-73
I112a	0.983	0.38	−1.373554963	2.90E-05
I117a	1.01	0.2	−2.35009599	2.08E-09
I117f	1.47	0.466	−1.672699662	6.93E-09
Illb	463.503	185.713	−1.304860538	0
1122	0.583	0.156	−2.004321154	8.51E-05
Ly6g	1.643	0.333	−2.280201881	9.13E-11
Ptes	26.136	11.82	−1.134363715	3.58E-277

*Gene is the gene name, FPKM is the average expression level, and log2 (PEG/IMQ) < 0 represents the gene down-regulated in the PEG group compared with the IMQ group*.

### PEG Ointment Reduces the Expression Level of Inflammatory Factor mRNA in the Epidermis of the Psoriasis Mouse Model

**Figure 5:**
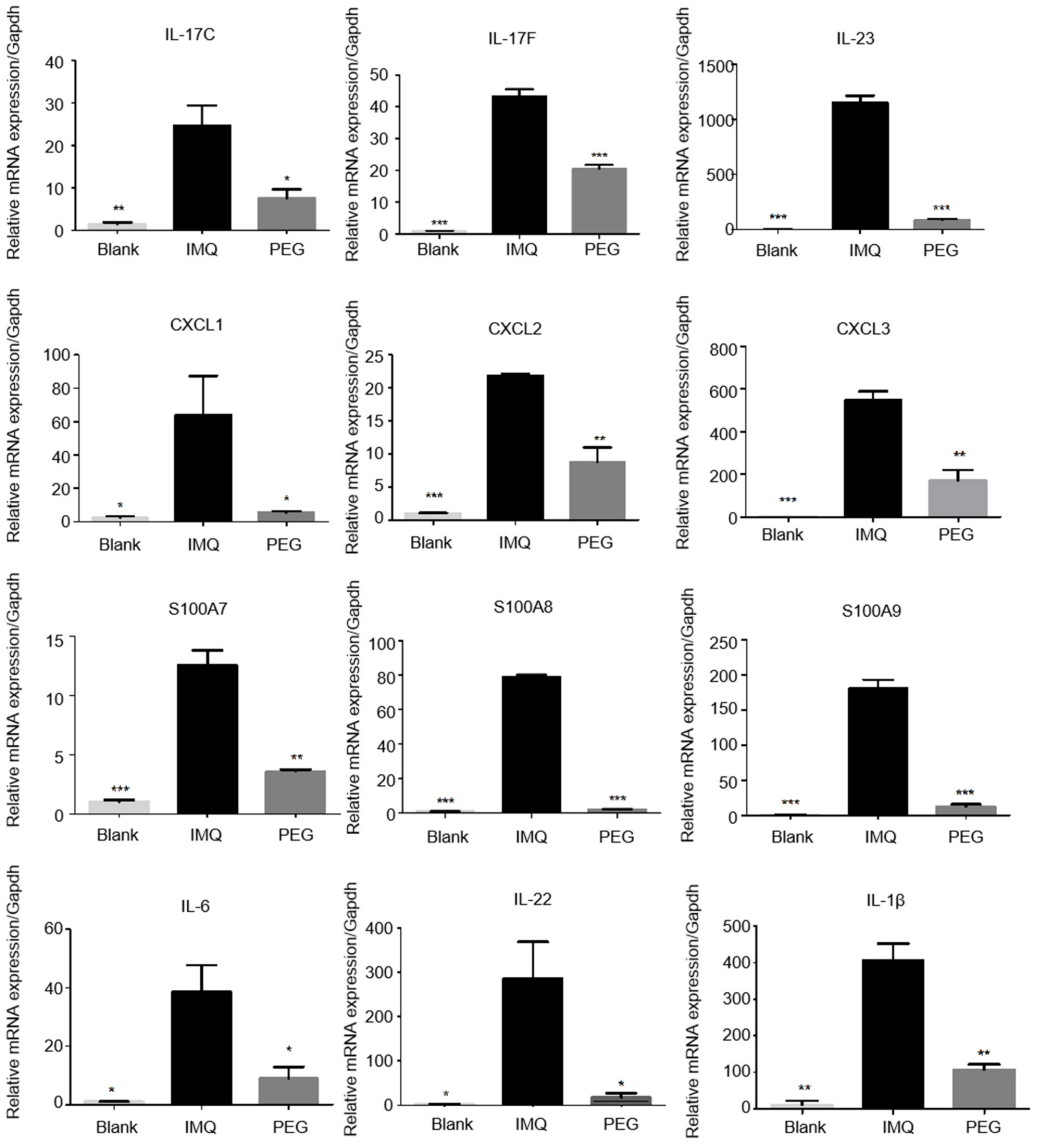
The mRNA expression levels of IL-23, IL-17C, IL-17F, IL-22, S100A7, S100A8, S100A9, CXCL1, CXCL2, CXCL3, IL-1β, and IL-6 in the skin lesions of each group (*compared to the IMQ group, *P < 0.05, **P < 0.01, ***P < 0.001). We have repeated the experiment more than three times.

The results of RNA sequencing suggested that the genes related to the functions of Th17 cells and MDSCs were significantly down-regulated. Therefore, we detected the relative mRNA levels of these genes of Th17 cells and MDSCs in the lesional tissues by Q-PCR. The results showed that PEG ointment could down-regulate the mRNA levels of genes IL-23, IL-17C, IL-17F, IL-22, S100A7, S100A8, S100A9, CXCL1, CXCL2, CXCL3, IL-1β, and IL-6 induced by the psoriasis mouse model (Figure 5).

### PEG Ointment Down-Regulates the Number of Immune Cells in the Spleen of Psoriasis Mice

**Figure 6:**
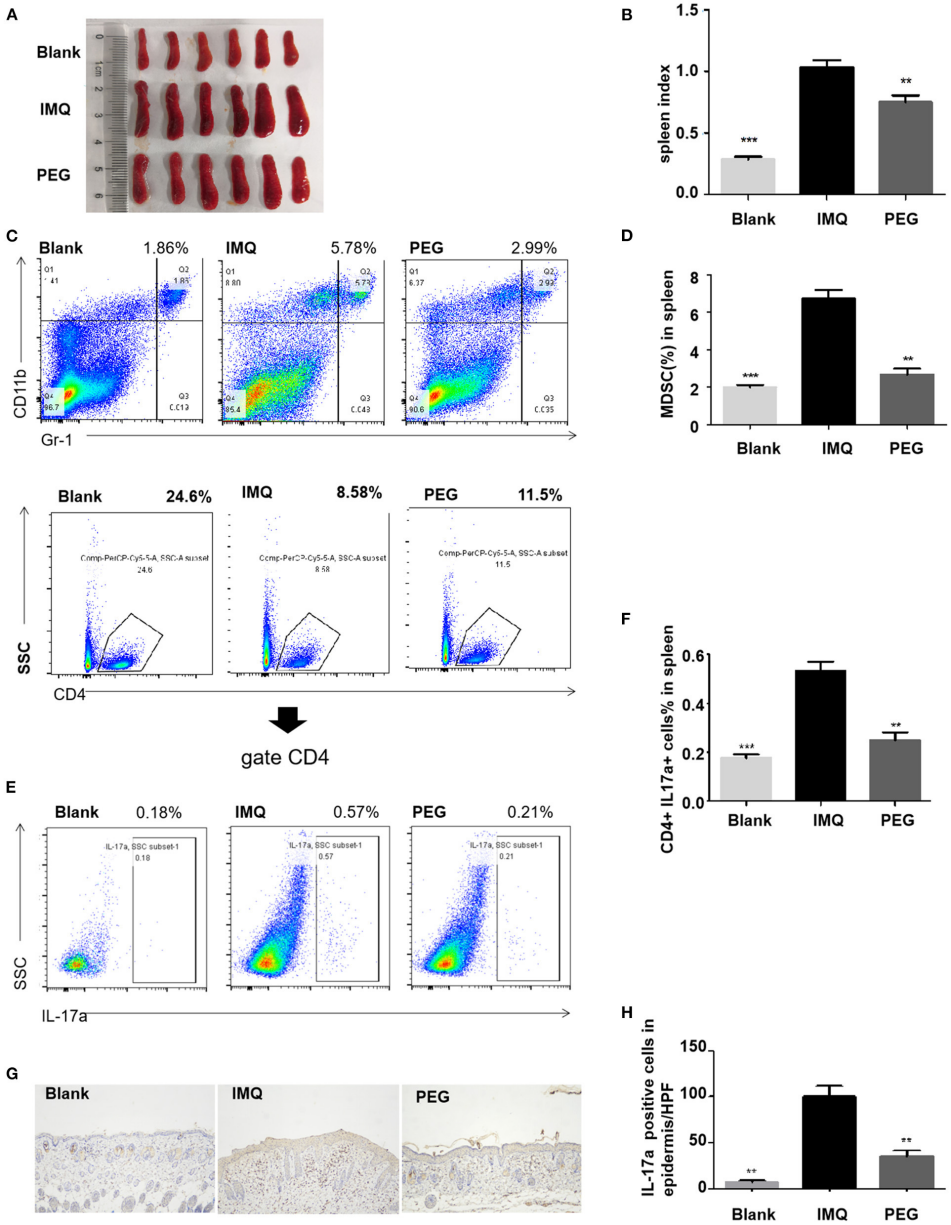
**(A,B)** Spleen and statistical graph of all mice. **(C–F)** Flow analysis chart of spleen and statistical graph. **(C,D)** Percentage of MDSCs in the spleen of each group of mice and statistical graph. **(E,F)** Percentage of Th17 cells in the spleen of each group of mice and statistical graph. **(G,H)** Infiltration of IL-17A cells in dermal superficial of each group of mice and statistical graph. (*compared with the IMQ group, **P < 0.01, ***P < 0.001). All statistical graph and data analysis were finished by GraphPad Prism 5 software. We have repeated the experiment more than three times.

Psoriasis is an immune-mediated disease, which is closely related to the IL-23/Th17 axis. In clinical practice, anti-IL-17 treatment can significantly improve psoriasis. The IMQ-induced psoriasis mice are also related to the IL-23/Th17 axis. We found that PEG ointment improved the signaling pathway in the psoriasis mice associated with Th17 cells and MDSCs by RNA sequencing. The results of Q-PCR were consistent with that of RNA sequencing, so we further analyzed the numbers of Th17 cells and MDSCs in the spleen by flow cytometry. It was found that PEG ointment could reduce the spleen index of the psoriasis mouse model (Figures 6A,B). In addition, the numbers of MDSCs and Th17 cells were increased in the spleen of the psoriasis mouse model compared with the Blank group. However, the numbers of MDSCs and Th17 cells were down-regulated after the treatment of PEG ointment (Figures 6C–F). Meanwhile, we performed immunohistochemical staining for the dermal cytoplasmic anti-IL-17A antibody in order to compare the Th17 cells infiltration inside the dermis among various groups (Figures 6G,H). It was found that the number of IL-17A cells with a positive expression increased significantly in the IMQ group compared to that in the Blank group and the number of IL-17A cells with a positive expression decreased significantly in the PEG group compared to that in the IMQ group. These results suggest that PEG ointment may improve the IMQ-induced psoriasis inflammation by reducing the numbers of Th17 cells and MDSCs, but the specific mechanism warrants further research.

## Discussions

Psoriasis is a disease that requires lifelong treatment, which imposes a great economic burden to the affected patients (14–16). At present, the first-line external medicine for the clinical treatment of psoriasis includes steroids, vitamin D_3_ derivatives, retinoic acid, and calcineurin inhibitors (17–20), which can inhibit the proliferation and differentiation of keratinocytes, the activation of T cells, and the release of inflammatory factors. In recent years, a growing number of studies have focused on the development of new drugs for psoriasis, including JAK inhibitors, Stat3 inhibitors, PDE4 inhibitors, TRK inhibitors, and AhR agonists (21–24). However, there was no report yet on the treatment of PEG. The pharmaceutical application of PEG mainly targets at the modification of PEG, that is, the combination of PEG with therapeutic protein drugs. PEGylated drugs can effectively overcome some shortcomings of traditional drugs, such as improving the pharmacokinetic behavior, reducing the excretion and administration frequency of the drug to maintain a stable concentration in the blood, and releasing the drug at the targeted site (25–27). PEGylated protein drugs have significantly improved the treatment outcomes of several chronic diseases such as hepatitis C, leukemia, severe combined immunodeficiency disease, RA, and Crohn's disease (28).

In this study, we observed that PEG ointment was effective for the IMQ-induced psoriatic-like inflammation. The PASI scores showed that PEG ointment could significantly improve the appearance of psoriasis-like lesions such as erythema, scales, and epidermal thickening. The pathological tissue staining showed that PEG ointment could improve the IMQ-induced keratinocyte hyperkeratosis, parakeratosis, acanthosis thickening, and inflammatory cell infiltration. The immunohistochemical staining showed that PEG ointment could down-regulate the high expression of Ki67. The RNA sequencing showed that the genes IL-17A, IL-17F, IL-22, CXCL2, and Ly-6G, which are closely related to Th17 cells and MDSCs, were down-regulated in the PEG group. We verified our results by Q-PCR. Specifically, we detected the mRNA expression level of the factors related to Th17 cells and MDSCs and found that the mRNA expression levels of IL-17C, IL-17F, IL-22, S100A8, S100A9, CXCL1, CXCL2, IL-6, and IL-1β were down-regulated after the treatment of PEG ointment in the mice of the IMQ group. The flow cytometry experiments indicated that PEG ointment could down-regulate the numbers of MDSCs and Th17 cells of the psoriasis model induced by IMQ cream. The immunohistochemical staining also showed that PEG ointment could reduce the infiltration of Th17 cells inside the dermis of psoriasis mice. In short, PEG ointment could improve the IMQ-induced psoriatic-like inflammation by down-regulating the numbers of MDSCs and Th17 cells and the secretion of inflammatory factors.

MDSCs are a group of heterogeneous cells from bone marrow origin. As the precursor cells of dendritic cells (DCs), macrophages, and granulocytes, MDSCs can significantly suppress immune cell responses. It is well known that the number of MDSCs in cancer patients is higher than that in the healthy population (29–31). This is because MDSCs in the peripheral blood increase and accumulate locally in the tumor and will release active substances to suppress the immune response and promote tumor growth. MDSCs are generally considered to be a poor prognosis signal for cancer (32), and what roles MDSCs play in psoriasis is of a great concern. It has been reported that MDSCs in the peripheral blood of psoriasis are increased and exhibit an immunosuppressive effect; however, the specific mechanism has not been clarified (33, 34). In recent years, the relationship between MDSCs and Th17 cells has been studied by many researchers. In tumor and rheumatoid arthritis (RA), the increase in the number of MDSCs is usually accompanied by the increase of Th17 cells. Zhang et al. reported that MDSCs promoted the differentiation of naïve CD4^+^ T cells into Th17 cells in an IL-1β-dependent manner (35). Wen et al. (36) found that MDSCs could secrete IL-1β to directly or indirectly promote the proliferation and differentiation of Th17 cells and also secrete IL-6 and TGF-β to indirectly promote the differentiation of naïve CD4^+^ T cells into Th17 cells. However, the interaction between MDSCs and Th17 cells still needs to be further explored.

It is well known that the IL-23/IL-17 axis is the key pathway leading to the pathogenesis of psoriasis. In the dermis, the IL-23 secreted by DCs induces the activation of Th17 cells, which subsequently releases various cytokines including IL-17A, IL-17F, and IL-22 to promote epidermal hyperplasia (37, 38). In addition, Th17 cells can also induce keratinocytes to produce IL-8 and antimicrobial peptides (e.g., S100A8 and S100A9) for the recruitment of neutrophils, the activation of vascular endothelial growth factor, and angiogenesis (39). Combined with our experimental results, we speculated that the number of MDSCs and the secretion of IL-1β and IL-6 in the psoriasis mice would decrease after treatment with PEG ointment, the ability of MDSC to promote Th17 proliferation and differentiation would be weakened, and, consequently, the Th17 function would be down-regulated. Eventually, the inflammatory reaction of psoriasis mice was alleviated. PEG ointment may improve the psoriasis-like inflammation by down-regulating the functions of Th17 cells and MDSCs.

## Conclusion

PEG ointment could improve the IMQ-induced psoriasis-like inflammation by down-regulating the functions of Th17 cells and MDSCs.

## Data Availability Statement

The original contributions presented in the study are included in the article/Supplementary Material, further inquiries can be directed to the corresponding author/s.

## Ethics Statement

The animal study was reviewed and approved by the Institutional Animal Care and Use Committee of Central South University, China.

## Author Contributions

Thanks to all authors for their contributions to this work. WZ provided the funding. Y-HK designed the research, modified the manuscript, and also provided some funding. YLu performed the research, analyzed data, and wrote the manuscript. YX helped with statistical analysis. M-ZY participated in the design of the research. X-CZ instructed the production of ointment. L-SW, W-QC, and YLuo also made some contributions to the research.

## Conflict of Interest

The authors declare that the research was conducted in the absence of any commercial or financial relationships that could be construed as a potential conflict of interest.
