# Supplementary figures and images for: Polyethylene Glycol Ointment Alleviates Psoriasis-Like Inflammation Through Down-Regulating the Function of Th17 Cells and MDSCs
Source: Front Med (Lausanne). 2021 Mar 22;7:560579. doi: 10.3389/fmed.2020.560579 (PMC8022287; doi:10.3389/fmed.2020.560579)

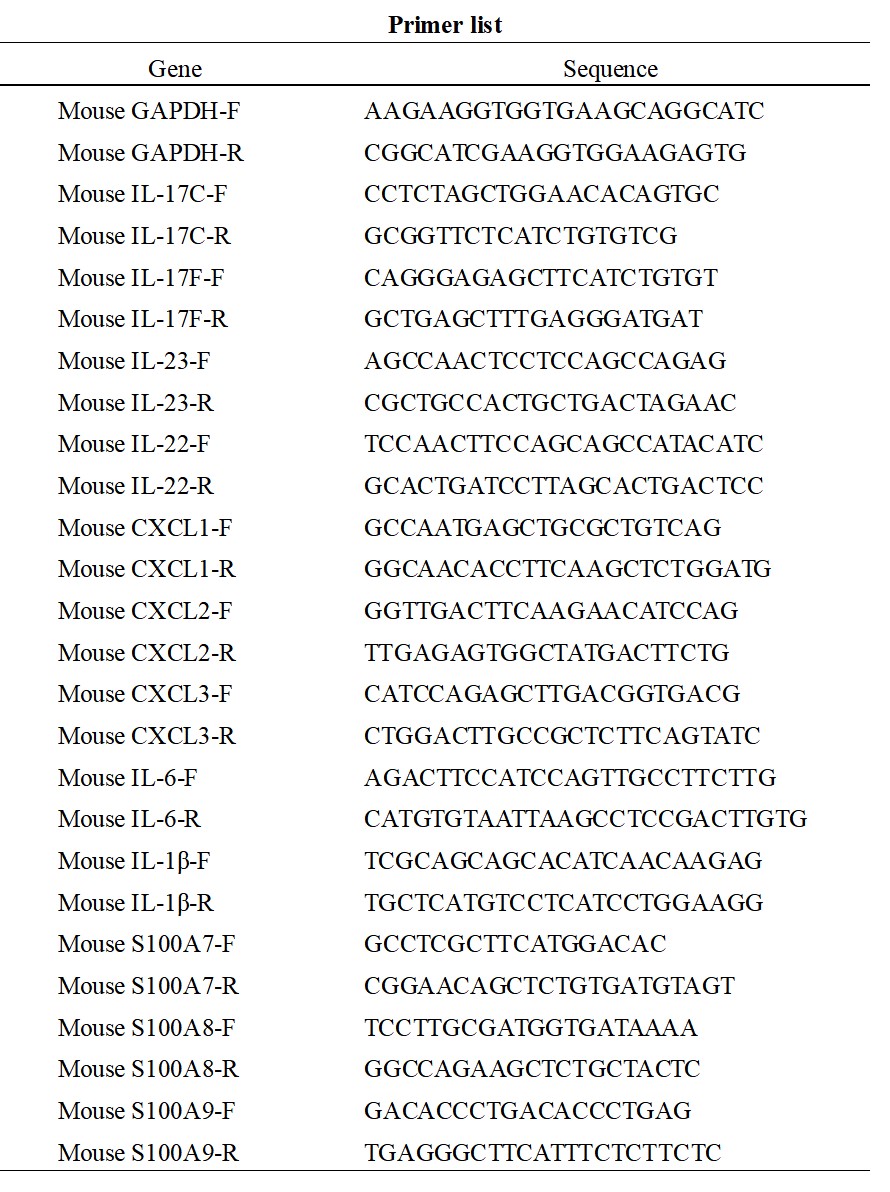

Supplement: Supplementary file 1 [file Image_1.jpeg]
